# C1q Regulates Horizontal Cell Neurite Confinement in the Outer Retina

**DOI:** 10.3389/fncir.2020.583391

**Published:** 2020-10-16

**Authors:** Courtney A. Burger, Danye Jiang, Fenge Li, Melanie A. Samuel

**Affiliations:** ^1^Department of Neuroscience, Baylor College of Medicine, Houston, TX, United States; ^2^Huffington Center on Aging, Baylor College of Medicine, Houston, TX, United States

**Keywords:** complement, C1q, retina, horizontal cells, microglia

## Abstract

During development, neurons generate excess processes which are then eliminated in concert with circuit maturation. C1q is the initiating protein in the complement cascade and has been implicated in this process, but whether C1q-mediated elimination is targeted to particular neural compartments is unclear. Using the murine retina, we identify C1q as a specific regulator of horizontal cell neurite confinement. Subsets of horizontal cell dendritic and axonal neurites extend into the outer retina suggesting that complement achieves both cellular and subcellular selectivity. These alterations emerge as outer retina synapses become mature. *C1q* expression is restricted to retina microglia, and the loss of C1q results in decreased microglia activation. This pathway appears independent of the C3a receptor (C3aR) and complement receptor 3 (CR3), as horizontal cells are normal when either protein is absent. Together, these data identify a new role for C1q in cell and neurite-specific confinement and implicate microglia-mediated phagocytosis in this process.

## Introduction

The complement pathway is comprised of over 20 innate immune signaling proteins. Work over the past 15 years has established that the central nervous system (CNS) can leverage complement to control synapse elimination during development and disease (Stevens et al., 2007; Chu et al., 2010; Bialas and Stevens, 2013; Hong et al., 2016). Deletion of C1q, the initiating complement pathway signaling molecule, results in delayed refinement of the dorsolateral geniculate nucleus of the thalamus and causes defects in the development of spinal motor circuits through altered microglia-mediated removal of excess neurites and synapses (Stevens et al., 2007; Vukojicic et al., 2019). C1q-mediated synapse removal has also been implicated in Alzheimer’s disease and in cognitive defects following neurotropic virus infection (Hong et al., 2016; Kunnakkadan et al., 2019). However, recent data suggest that complement may be selective for particular CNS regions. For example, C1q is dispensable for developmentally regulated ocular dominance plasticity in the visual cortex (Welsh et al., 2020). Thus, C1q may target particular neuronal circuits or even specific neuron or synapse types for elimination.

Surprisingly little is known about whether or how complement is regionally regulated in the CNS. Since circuit specificity is critical for neural function, solving this mystery is an important goal. Classical complement signaling triggers a protease cascade, leading to the deposition and cleavage of downstream complement proteins that in turn mediate a host of diverse responses (Ricklin et al., 2010). However, comparatively little is known about the underlying cell or neurite type-specific mechanisms that control this process. Progress toward this goal has been limited in part by neuron and synapse heterogeneity in the brain, where diverse neuron types are intermingled and their identities are often unknown.

**FIGURE 1:**
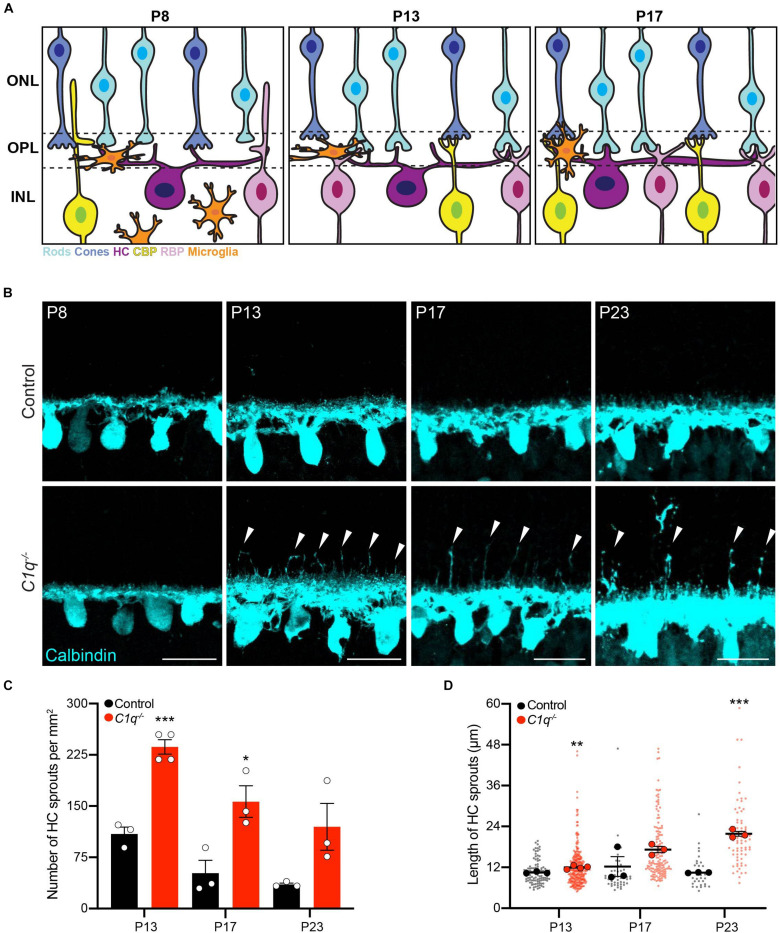
C1q regulateshorizontal cell neurite confinement. **(A)** Schematic of outer retina neuron and microglia organizationduring the second postnatal week. The outer nuclear layer (ONL) contains cones (blue) and rods (cyan) that form connections in the outer plexiform layer (OPL) with horizontal cells (purple), rod bipolar cells (pink), and cone bipolar cells (yellow) located in the inner nuclear layer (INL). At P8, microglia are migrating toward the OPL in concert with cone synapse maturation and rod synapse formation. At P13, microglia reside in the OPL, eye opening occurs, and ribbon synapse maturation continues as outer retina synapses become active. At P17, microglia remain in the OPL as sublamination of rod and cone synapses is completed. **(B–D)** Representative images of horizontal cells (**B** anti-calbindin, cyan) and quantification of ectopic horizontal cell neurites (**C,D**) in wild type control and *C1q^–/–^* mice at P8, P13, P17, and P23. Horizontal cells in *C1q^–/–^* mice fail to remain confined to the OPL, showing an increased number of ectopic neurites (arrowheads) that peak at P13 but remain present as development proceeds (**C**, *N* ≥ 3 wild type control and *C1q^–/–^* mice). These neurites extend into the outer nuclear layer and grow longer over time (**D**, *n* = 99 and 287 sprouts from three wild type control and four *C1q^–/–^* mice at P13; *n* = 47 and 142 sprouts from three wild type control and three *C1q^–/–^* mice at P17; *n* = 32 and 73 sprouts from three wild type control and three *C1q^–/–^* mice at P23). Scale bars = 25 μm. Data are represented as the mean ± SEM **(C)** or as beeswarm SuperPlots **(D)** in which individual horizontal cell sprout values are presented together with the mean from each animal ± the SEM. ***p < 0.001, **p < 0.01, *p < 0.05 using unpaired two-tailed Student’s *t*-test.

To begin to shed light on these questions, we focus here on the murine retina. In this system, neurons are layered and organized, and their identities and connectivity are well-mapped. Connectivity is further simplified in the outer retina where just two synapse types occur between four well-defined neural partners: rods, cones, horizontal cells, and bipolar cells (Dacheux and Raviola, 1986; Ghosh et al., 2004; Zhang et al., 2006). These neuron types display synaptic specificity: rods connect mainly with horizontal cell axons and rod bipolars while cones connect with horizontal cell dendrites and cone bipolars. The outer retina synapse layer begins to emerge at P5, but these connections are not functionally active until P14 when interneurons are integrated and eyes are open (Figure 1A). The retina also contains microglia whose localization to retinal synaptic layers coincides precisely with the period of synapse maturation and pruning (reviewed in Li F. et al., 2019). Deletion of C1q and other complement components can reduce retina function in adult and aged animals (Mukai et al., 2018, Supplementary Figure 1), but the developmental impacts of complement on the retina are largely unknown.

Using the retina, we show that loss of C1q leads to marked and specific defects in developmental horizontal cell neurite confinement. Subsets of horizontal cell neurites arising from both dendrites and axons extend into the outer retina, suggesting that complement can selectively target particular cell types. We further show that *C1q* expression in the developing retina is restricted to microglia, and C1q mutants show decreased microglia activation. Genetic ablation of C3a receptor (C3aR) and complement receptor 3 (CR3) do not cause similar alterations, suggesting that C1q-mediated regulation of horizontal cell neurites may be independent of these pathways. These data identify a new role for C1q in the regulation of specific cellular and subcellular compartments via microglia activation in the retina.

## Materials and Methods

### Animals

The *C1q^–/–^* strain has been described previously (Botto et al., 1998) and was provided by Dr. Farrah Kheradmand, Baylor College of Medicine, who received them with permission from Dr. Marina Botto, Imperial College London. In this strain, the *C1qa* gene was disrupted by the insertion of a neomycin-resistance cassette in the coding region of the first exon. These mice were backcrossed to C57BL/6NJ mice, and wild type mice of this strain were used as controls (Jackson labs stock #005304). This background contains the *rd8* mutation, but this does not impact retinal organization or horizontal cell neurite restriction within the time frame of this analysis (Albrecht et al., 2018). The *C3aR^–/–^* (Humbles et al., 2000, Jackson labs stock #00512) and the *CR3^–/–^* (Coxon et al., 1996; Jackson labs stock #003991) strains have been described previously and were provided by Dr. Hui Zheng and Dr. Farrah Kheradmand, Baylor College of Medicine. The *C3aR^–/–^* mice were backcrossed to C57BL/6J (Jackson labs stock #000664). Both C57BL/6J and *CR3*^+/^*^–^* littermates were used as controls. Experiments were carried out in both male and female mice in accordance with the recommendations in the Guide for the Care and Use of Laboratory Animals of the NIH under protocols approved by the BCM Institutional Animal Care and Use Committee.

### Immunohistochemistry

Eyes were collected from P5, P8, P10, P13, P17, and P23 mice. The day of birth was designated as postnatal day 0 (P0). Whole eyes were fixed for 45 min in 4% PFA and then rinsed with PBS. Retina cross-sections and flat mounts were prepared as previously described (Samuel et al., 2014). Briefly, for cross-section analysis, eyecups were dissected by removing the cornea and lens. The eyecups were cryoprotected in 30% sucrose, embedded in OCT compound (VWR), stored at −80°C, and then sectioned at 20 μm. For microglia cross-section staining, 100 μm vibratome sections were prepared from retina embedded in 6% agarose. For both cryostat and vibratome cross-sections, slices were incubated with blocking solution (3% normal donkey serum and 0.3% Triton X-100 in PBS) for 1 h, and then with primary antibodies (Table 1) O/N at 4°C. The following day, slides were washed three times with PBS for 10 min each and incubated with secondary antibodies (Jackson ImmunoResearch Laboratories) for 1 h at room temperature. Slides were then washed again and mounted in Vectashield (Vector Laboratories). For flat-mount preparations, the retina was removed from the eyecup and incubated in blocking solution (10% normal donkey serum and 0.5% Triton X-100 in PBS) for 1 h before proceeding with primary antibodies (Table 1) for 3 days followed by washes with PBS and secondary antibodies for 2 days at 4°C. Images were acquired on an Olympus Fluoview FV1200 confocal microscope and processed using FIJI.

**TABLE 1:** Antibodies used in tissue analysis.

Antibody name	Immunogen	Labeling specificity	Source	Concentration
Arrestin	Synthetic linear peptide	Cone photoreceptors	Millipore; rabbit polyclonal; AB15282; RRID:AB_11210270	1:5000
Calbindin D-28k	Full-length recombinant human Calbindin D-28K	Horizontal cells; amacrine cells; retinal ganglion cells	Novus biologicals; chicken polyclonal; NBP2-50028; no RRID	1:2000
Calbindin D-28k	Recombinant rat calbindin D-28k (CB)	Horizontal cells; amacrine cells; retinal ganglion cells	Swant; rabbit polyclonal; CB38; RRID:AB_10000340	1:10,000
CD68	Purified concanavalin A acceptor glycoprotein from P815 cell line	Lysosomes	Bio-Rad; rat monoclonal; MCA1957; RRID:AB_322219	1:500
Iba1	Synthetic peptide (C-terminal of Iba1)	Microglia	Wako; rabbit polyclonal; 019-19741; RRID_AB:839504	1:500
OPN1SW	Peptide mapping at the N-terminus of the opsin protein encoded by OPN1SW of human origin	Cone photoreceptors	Santa Cruz; goat polyclonal; sc-14363; RRID:AB_2158332	1:500
Protein Kinase C alpha (PKCa)	Purified bovine brain protein kinase C	Rod bipolar cells	Abcam; mouse monoclonal; ab31; RRID:AB_303507	1:500
PSD95	Synthetic peptide corresponding to Mouse PSD95 aa 1-100 (C-terminal) conjugated to keyhole limpet hemocyanin.	Photoreceptor terminals	Abcam; goat polyclonal; ab12093; RRID:AB_298846	1:500
Ribeye	Recombinant protein corresponding to AA95 to 207 from rat Ribeye	Ribbon synapses	Synaptic Systems; rabbit polyclonal; 192 103; RRID:AB_2086775	1:500
Secretagogin (SCGN)	Recombinant human secretagogin	Cone bipolar cells	BioVendor Laboratory Medicine; rabbit polyclonal; RD181120100; RRID:AB_2034060	1:500
VGlut1	Recombinant protein corresponding to AA456 to 560 from rat VGLUT1	Photoreceptor terminals	Syanptic Systems; rabbit polyclonal; 135 302; RRID:AB_887877	1:500

*Antibodies were used to label individual neuron populations and synaptic proteins in the outer retina.*

### Whole Retina qRT-PCR

Retinas were dissected in ice-cold RNase-free water, and each pair of retinas were homogenized separately. Total RNA was purified from each sample using a RNeasy Plus Mini Kit according to the manufacturer’s instructions (Qiagen). First strand cDNA synthesis was performed using a complementary DNA synthesis kit according to the manufacturer’s protocol (iScript Reverse Transcription Supermix for qRT-PCR; Bio-Rad Laboratories, Inc.). Quantitative real-time PCR (qRT-PCR) was performed with primers to targets and house-keeping genes (for primers, see Table 2) using iTaq Universal SYBR Green Supermix (Bio-Rad) and a CFX384 Touch Real-Time PCR Detection System (Bio-Rad). Relative quantification was determined using the ΔΔC_t_ method (Livak and Schmittgen, 2001). Genes of interest were normalized to *GAPDH*. Primers were designed in-house using the Primer-BLAST software or obtained from the Harvard Primer Bank and others (Spandidos et al., 2010; Silverman et al., 2019).

**TABLE 2:** qRT-PCR primers used in mRNA analysis.

Gene name	Sequence	Sources
C1qa	AAAGGCAATGCAGGCAATATCA	Harvard Primer Bank (Spandidos et al., 2010)
	TGGTTCTGGTATGGACTCTCC	
C3aR	GGAAGCTGTGATGTCCTGG	Silverman et al., 2019
	CACACATCTGTACTCATATTGT	
CR3	GCAGGAGTCGTATGTGAGG	Silverman et al., 2019
	TTACTGAGGTGGGGCGTCT	
CD68	GGACCCACAACTGTCACTCAT	Harvard Primer Bank (Spandidos et al., 2010)
	AAGCCCCACTTTACC	
PSD95	TGAGATCAGTCATAGCAGCTACT	Designed in house
	CTTCCTCCCCTAGCAGGTCC	
VGlut1	GGTGGAGGGGGTCACATAC	Harvard Primer Bank (Spandidos et al., 2010)
	AGATCCCGAAGCTGCCATAGA	
Syt2	AGAACCTGGGCAAATTGCAGT	Harvard Primer Bank (Spandidos et al., 2010)
	CCTAACTCCTGGTATGGCACC	
SYP	CAGTTCCGGGTGGTCAAGG	Harvard Primer Bank (Spandidos et al., 2010)
	ACTCTCCGTCTTGTTGGCAC	
GAPDH	AGGTCGGTGTGAACGGATTTG	Harvard Primer Bank (Spandidos et al., 2010)
	TGTAGACCATGTAGTTGAGGTCA	

### RNAscope

RNAscope was performed using Probe-Mm-C1qa (Cat. # 441221-C2, ACD-bio) on 20 μm retina sections collected as described above for immunohistochemistry. The commercially available RNAscope fluorescent multiplex assay was performed according to the manufacturer’s instructions (ACD-bio) with the following modifications. Tissue samples were dehydrated using an ethanol gradient of 10, 30, 50, 70, and 100% (3 min each), and the boiling time in target retrieval solution was reduced to 5 min. After RNAscope, slides were co-stained with Iba1 and calbindin to visualize microglia and horizontal cells, respectively.

### Electroretinography

We performed ERG on 2-month-old adult mice as previously described (Albrecht et al., 2018). In brief, mice were dark adapted overnight and anesthetized with 1.5% isoflurane at an oxygen flow rate of 1.0 L/min. Mice were placed on a heated platform on the Diagnosys Celeris ERG system (Diagnosys), and pupils were dilated using phenylephrine hydrochloride and tropicamide, followed by Gonak solution. A contact lens-style electrode was placed on the eye to record electroretinograms. A ground electrode was placed subcutaneously into the haunch of the animal, while a reference electrode was placed in the forehead of the animal. The Diagnosys Celeris ERG system was used to elicit both scotopic and photopic responses. Scotopic responses were elicited in the dark with flashes ranging from 0.003 to 20.0 cd^∗^s/m^2^. Photopic responses were elicited after eyes had been adapted to light for 5 min at an intensity of 3.0 and 10.0 cd^∗^s/m^2^.

### AAV Mediated Single-Cell Neuron Labeling and Reconstruction

For AAV-mediated neuron labeling, P4 *C1q*^–/–^ and control pups were anesthetized on ice. To label individual horizontal cells, the sub-retinal and intravitreal space were inoculated with AAV2/9CMVCre-wtIRESeGFP (viral stocks generated by the Viral Vector Core Facility at University of Iowa). Mice were injected subretinally using a 1:500 viral dilution and sacrificed at P23. To visualize singly labeled cells, retinas were processed for flat-mount preparation as described above following staining with an anti-GFP antibody to label targeted neurons and anti-calbindin to label horizontal cells. Axons and dendrites from the same cells were imaged in a 635.9 μm x 635.9 μm field on an Olympus Fluoview FV1200 confocal microscope at a step size (Z) of 0.5 μm. The images were then imported into IMARIS (Bitplane) to create 3D surface rendering. 0.2 μm smoothing was used for GFP-positive and calbindin-positive horizontal cells.

### Histological Quantification

All quantifications were performed using retinal sections prepared from *C1q^–/–^, C3aR^–/–^, CR3^–/–^*, and age-matched control animals. C57BL/6NJ, C57BL/6J or littermate controls were used in all experiments, and all images were acquired at equivalent retinal eccentricities from the optic nerve head. For all experiments, data were collected from three to five mice per group, and three to four images per animal were obtained. To quantify ectopically localized horizontal cell neurites, a calbindin antibody was used to label horizontal cells and DAPI was used to visualize the nuclei. An ectopic neurite was quantified as a neurite that extended at least one nucleus above the lower boundary of the ONL, and the length of these neurites were measured using FIJI. To assess horizontal cell density and mosaic formation, the location of each calbindin-positive horizontal cell was recorded in a 635.9 μm × 635.9 μm image field sampled at > 3 locations in each animal. The XY coordinates of horizontal cells were then used to calculate the Voronoi domain regularity index, density recovery profile, and cell density using FIJI and WinDRP (Rodieck, 1991). To quantify retinal layer thickness, DAPI was used to label nuclei, and the length of each layer was measured. The number of microglia process endpoints and the summed branch length per microglia in the OPL were quantified as previously described (Young and Morrison, 2018). In brief, each image was skeletonized after optimization and turned into a binary image. The Analyze Skeleton Plugin was then run in FIJI. Fragments less than 1.7 μm were removed from analysis, and the individual endpoint and branch length were summed and divided by the total number of microglia. The number of microglia in the OPL was determined by counting the total number of cells labeled with the microglia specific antibody Iba1.

### Quantification of Microglial Activation State

Immunohistochemistry was performed on flat mount P10 retinas with antibodies against Iba1 and CD68 (Table 1) as described above. For each genotype, *N* ≥ 3 animals were imaged. Three 211.97 μm × 211.97 μm images were sampled per animal and were acquired on a confocal microscope (60X objective, 1.5X zoom) using a step size (Z) of 0.5 μm. The images were then processed and analyzed using FIJI and IMARIS (Bitplane) as previously described (Schafer et al., 2014). In brief, background was subtracted from both fluorescent channels at a rolling ball radius of 10, and a mean filter was applied to the Iba1 channel at 1.5. Processed image stacks were then uploaded into IMARIS to create 3D volume surface renderings and were used to determine the volume of the Iba1-positive microglia and the volume of CD68 staining. To measure the volume, any CD68 signal outside the Iba1-positive microglia was masked in the image using the mask function. The remaining fluorescence within the microglia was then surface rendered and total volume of CD68 staining was calculated. To determine percent volume of CD68 staining, the volume of the internal CD68 (μm^3^) was divided by the volume of the Iba1-positive microglia (μm^3^). All analyses were performed blind to the genotype.

### Statistical Analysis

Analyses of ERG results were performed using an unpaired *t*-test between groups at each flash intensity, with quantifications corrected for multiple comparisons using the Holm-Sidak method. Analyses of the number of horizontal cell sprouts, the length of horizontal cell sprouts, horizontal cell mosaic, retinal layer thickness, qRT-PCR, the number of process endpoints per microglia, the summed branch length per microglia, and the percent CD68 positive lysosome volume were performed using an unpaired two-tailed Student’s *t*-test. Statistical differences were evaluated using GraphPad Prism 8 software. *p* < 0.05 was considered statistically significant.

## Results

### C1q Regulates Horizontal Cell Confinement to the OPL

To examine the role of C1q in retina development, we obtained C1qa null mice in which the A chain of C1q is deleted, resulting in C1q loss-of-function (Botto et al., 1998). *C1q* was absent in the retina in these animals as confirmed by RNAscope and whole retina qPCR (Supplementary Figure 2). We assessed synapse layer emergence and neuron organization in the outer retina as these connections develop (P5–P17). In control animals, this process involves nascent outer plexiform layer (OPL) formation and reorganization of horizontal cell neurites from an apical to a lateral orientation at P5. This is followed by integration of bipolar cells at P8. Outer retina synapse maturation is complete at P14 when organized neuron terminals are restricted to the OPL and eye opening occurs (Figure 1A). Early synapse emergence events were indistinguishable in control and *C1q^–/–^* animals both at P5 as the OPL emerges and at P8 (Figure 1B and Supplementary Figure 3). However, as outer retina synapses became active, neuron-specific defects emerged in *C1q^–/–^* mice. Horizontal cells failed to remain confined to the OPL at P13 in *C1q^–/–^* mice, extending numerous long neurites into the outer retina (Figure 1B). This resulted in 236.5 horizontal cell sprouts per mm^2^, representing a 70.6% increase compared to controls (*p* = 0.0004, Figure 1C). *C1q^–/–^* dependent horizontal cell defects persisted during development, and these ectopic neurites grew longer over time. At P17 and P23 the average lengths of ectopic horizontal cell neurites in *C1q^–/–^* mice were 17.2 and 21.8 μm, respectively (Figure 1D). These defects did not alter horizontal cell spacing or mosaic distribution, suggesting that nuclear patterning is maintained while neurite specific organization is disrupted (Supplementary Figure 4). To examine the specificity of these alterations, we assessed the thickness of all retina layers together with the organization and neurite restriction of three other outer retina neuron types: cones, cone bipolar cells, and rod bipolar cells. The thickness of all retinal layers was unchanged in *C1q^–/–^* mice relative to controls, and no morphological differences were observed at P13 in the cellular arrangement, neurite organization, or synapse protein levels in these cells in the absence of C1q (Supplementary Figures 5, 6). Together, these data show that C1q is involved specifically in confining horizontal cell neurites to the OPL following eye opening.

**FIGURE 2:**
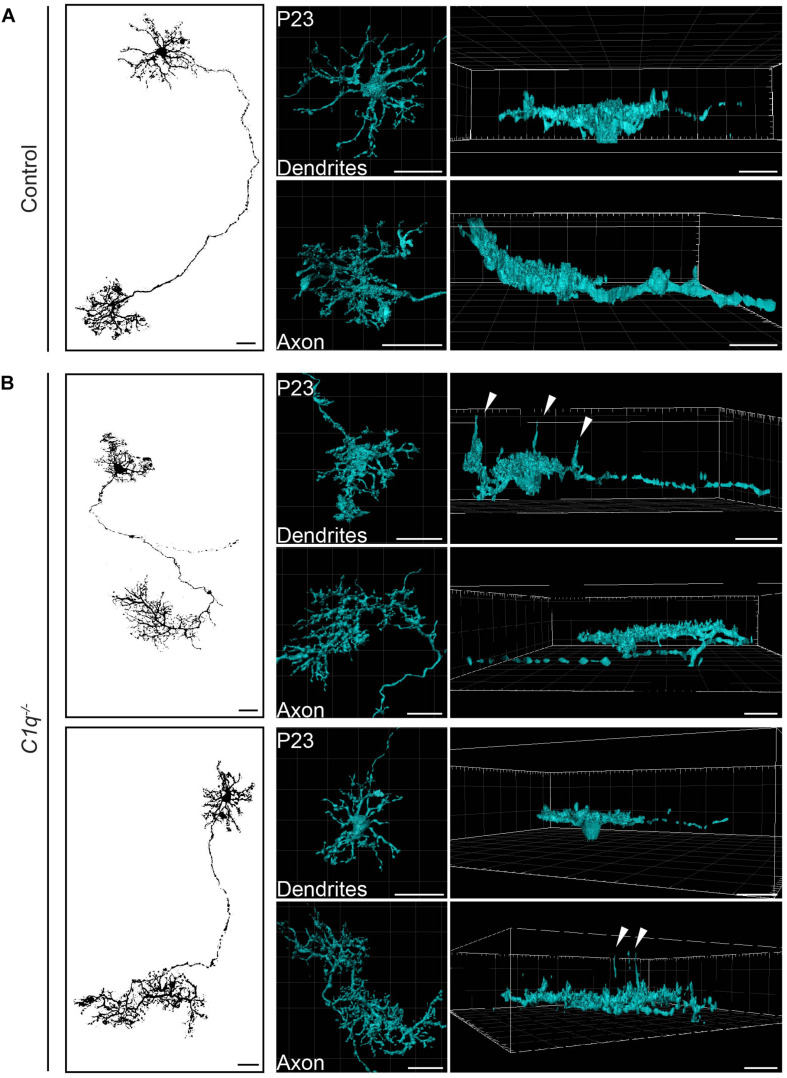
C1q confines subsets of horizontal cell neurites within axons and dendrites. Representative single AAV-labeled horizontal cells from wild type control (**A**) and *C1q^–/–^* (**B**) mice were reconstructed at P23. An en face image of each horizontal cell (left panel) is shown together with the individual reconstruction of that cell’s axon and dendrite presented in an en face (middle panel) and lateral view (right panel). Subsets of neurites from both axons and dendrites extend ectopically in the *C1q^–/–^* mice (arrowheads), while no such neurite extensions were observed in controls. *n* = 4 horizontal cells each from three wild type control and three *C1q^–/–^* mice, respectively. Scale bars = 25 μm.

Horizontal cells are highly polarized such that dendrites contact cones and axons contact rods. We thus asked whether C1q modulates the confinement of horizontal cell axons, dendrites, or both. To achieve this, we performed single horizontal cell labeling and reconstruction using AAV2/9-GFP. In control animals, both compartments were lateralized, with axons and dendrites restricted to a thin lamina (Figure 2A and Supplementary Videos 1, 2). This pattern differed in *C1q^–/–^* mice. Long extensions were observed arising from both horizontal cell compartments, with cells displaying axon neurite extensions (Figure 2B and Supplementary Videos 3, 4) and dendritic neurite extensions (Figure 2B and Supplementary Videos 5, 6). Only some neurites arising from a single horizontal cell were affected, suggesting that in addition to cellular specificity, C1q dependent remodeling may target distinct subcellular regions of a given neural compartment.

### Microglia May Restrict Horizontal Cells via C1q Mediated Phagocytosis

**FIGURE 3:**
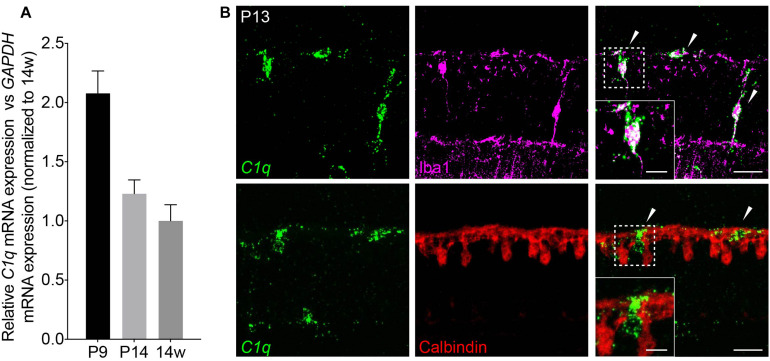
*C1q* is expressed in retina microglia during development. **(A)** qRT-PCR for *C1q* at P9, P14, and 14 weeks. Values represent the fold mRNA expression level relative to the levels detected in adult animals following normalization to *GAPDH*. **(B)** Representative fluorescent *in situ* hybridization images at P13 of *C1q* (green) co-stained with either Iba1 (magenta) or calbindin (red) to label microglia and horizontal cells, respectively. *C1q* colocalizes with microglia but not with horizontal cells in the outer retina at this time (arrowheads). Inset shows *C1q* expression in Iba1-positive microglia that is absent in calbindin-positive horizontal cells. Scale bars = 25 and 10 μm (inset).

Next, we investigated the cellular basis of C1q driven horizontal cell neurite remodeling. To begin, we examined the levels and localization of C1q over development in order to determine the peak of its expression and the cellular source from which it is derived. We performed qRT-PCR for *C1q* on whole retina at P9, P14, and in adult mice (Figure 3A). *C1q* gene expression was greatest at P9 but was also present at P14 at higher levels than in adult animals, consistent with ongoing retina synapse formation and remodeling at these time points. To identify the cellular source of *C1q*, we performed RNAscope at P13 together with co-staining using antibodies specific for microglia (Iba1) and horizontal cells (calbindin) (Table 1). We found that *C1q* expression was restricted predominately to microglia in the outer and inner retina (Figure 3B). These data suggest that microglia are the major cellular source of *C1q* when horizontal cell confinement phenotypes are observed.

**FIGURE 4:**
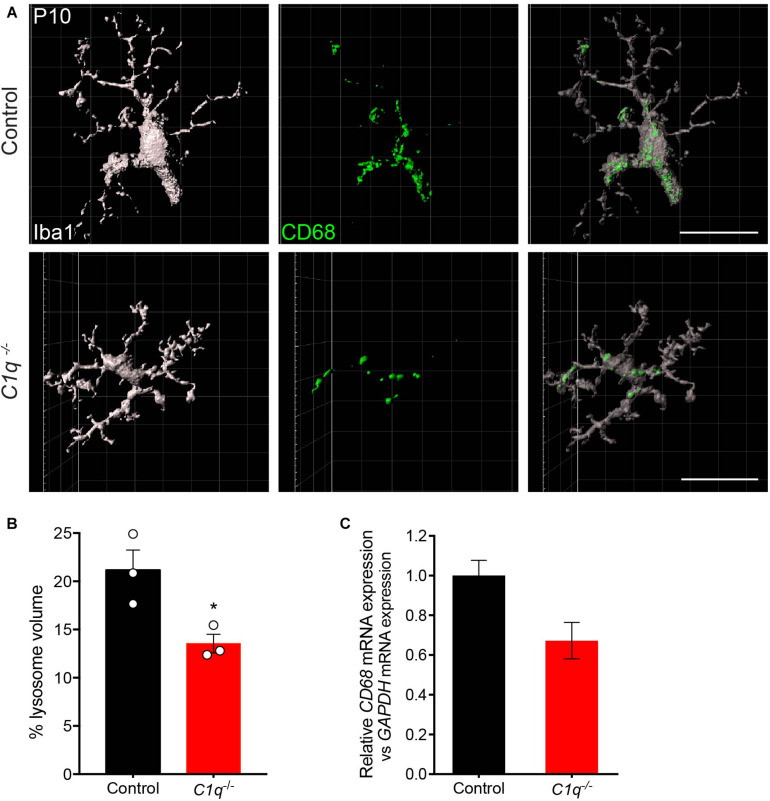
Loss of C1q leads to decreased microglia activation. **(A,B)** Retinas from *C1q^–/–^* and wild type control mice at P10 were stained for microglia (Iba1, gray) and the lysosome marker CD68 (green). Outer retina microglia were reconstructed **(A)** and used to quantify the relative lysosomal volume within individual cells **(B)**. Microglia from *C1q^–/–^* mice show a significant decrease in CD68-positive lysosomal volume relative to controls. **(C)** The levels of *CD68* mRNA were quantified in wild type control and *C1q^–/–^* mice using qRT-PCR and normalized to *GAPDH*. *CD68* mRNA levels trended lower but were not significantly different. *N* = 3 control and *N* = 3 *C1q^–/–^* mice. Scale bars = 25 μm. Data are represented as the mean ± SEM. *p < 0.05 using unpaired two-tailed Student’s *t*-test.

We then asked whether C1q deficiency altered microglia organization or activation. To assess this, we first examined microglia localization and morphology. At P13, microglia appeared to populate the outer retina synapse layer in C1q null mice at similar levels to that in controls, and no significant morphological differences were observed (Supplementary Figures 7A–D). To examine microglia activation, we assessed the levels of CD68 that colocalized with microglia. This marker labels lysosomes following phagocytosis and is present in highly activated microglia (da Silva and Gordon, 1999; Taylor et al., 2005). We imaged and reconstructed individual microglia in the outer synapse layer of the retina at P13 and assessed the mean percent volume of CD68 found within each cell (Figure 4A). C1q mutants displayed a significantly decreased volume of CD68 within microglia with 21.2% of the total microglia volume occupied in controls relative to 13.6% in mutants (*p* = 0.0302, Figure 4B). Consistent with this finding, the levels of *CD68* expression in whole retina appeared somewhat lower in *C1q^–/–^* mice relative to controls (Figure 4C). Together these data suggest that the loss of C1q leads to reduced microglia activation, which in turn may contribute to reduced microglia-mediated engulfment and confinement of ectopic horizontal cell processes.

### Horizontal Cell Confinement Occurs Independently of C3aR and CR3

**FIGURE 5:**
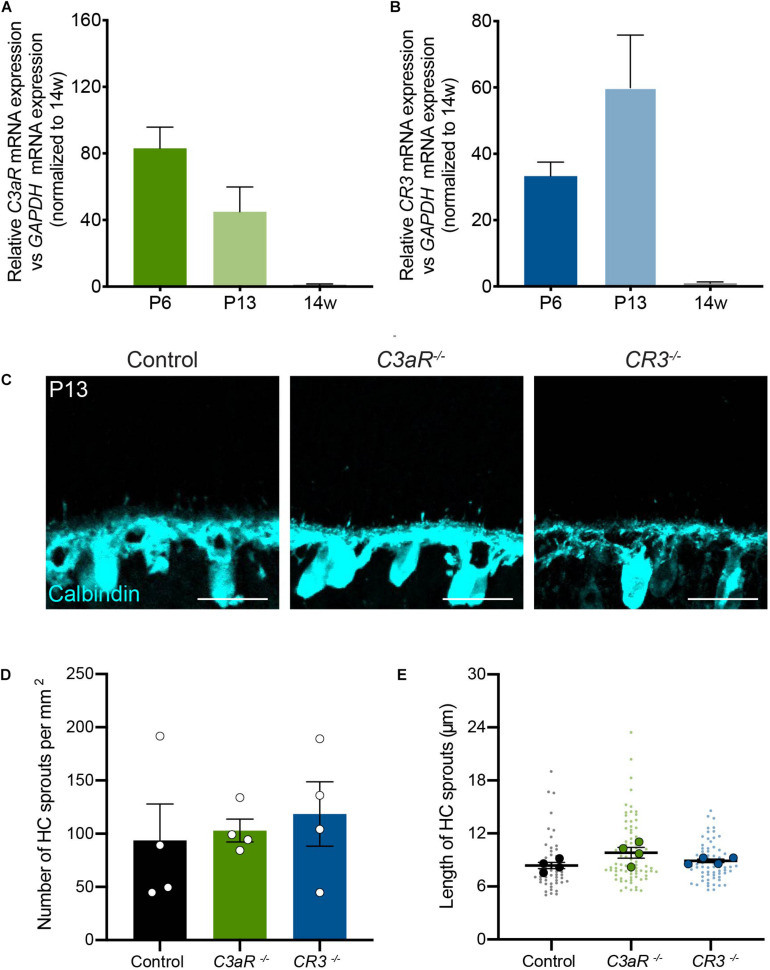
Horizontal cells develop normally with the loss of either C3aR or CR3. **(A,B)** qRT-PCR for complement proteins *C3aR* and *CR3* at P6, P13, and adult in wild type mice. Values represent the fold mRNA expression level relative to the levels detected in control animals following normalization to *GAPDH*. *N* = 3 animals. Representative images of horizontal cells (**C**, anti-calbindin, cyan) and quantifications **(D,E)** of ectopic horizontal cell neurites in control and *C3aR*^–/^*^–^* or *CR3^–/–^* mice at P13. Horizontal cell neurites in *C3aR*^–/^*^–^* and *CR3^–/–^* mice remain confined to the OPL, showing no increase in the number **(D)** or length **(E)** of ectopic neurites (*n* = 52 sprouts from four control mice; *n* = 84 sprouts from four *C3aR^–/–^* mice; *n* = 67 sprouts from four *CR3*^–/^*^–^* mice). Scale bars = 25 μm. Data are represented as the mean ± SEM **(D)** or as beeswarm SuperPlots **(E)** in which individual horizontal cell sprout values are presented together with the mean from each animal ± the SEM.

The complement pathway is comprised of over 20 signaling molecules, each of which is involved in distinct activation pathways. Among these, several recent studies have uncovered important roles for the C3 pathway, which is cleaved downstream of C1q, generating C3a and C3b. These effectors can participate directly in signaling that leads to the formation of the membrane attack complex (MAC), but they can also activate downstream signaling by binding to their receptors, which include C3aR and CR3, respectively. In particular, signaling through the C3-C3aR pathway has been implicated in excessive neuron loss in Alzheimer’s disease and in viral-induced synapse loss in experimental lupus models (Jacob et al., 2010; Lian et al., 2016; Vasek et al., 2016). We examined the levels of *C3aR* and *CR3* over development and in adults in order to determine the peak of their expression. The levels of both molecules were highest in the first two postnatal weeks during outer retina development (Figures 5A,B). We thus examined horizontal cell neurite confinement during development in C3aR and CR3 deficient mice. In both lines, horizontal cell confinement was unchanged (Figure 5C). *C3aR^–/–^* mice showed no significant difference in horizontal cell neurite localization, sprout number, or length relative to controls at P13, with short and rare ectopic neurites detected in both cases (*p* = 0.8065, Figures 5C–E). Similarly, horizontal cell neurites were restricted normally in *CR3^–/–^* mice relative to controls at P13 (*p* = 0.6066, Figures 5C–E). Together, these data suggest that C1q signals in microglia through CR3 and C3aR-independent pathways to properly confine horizontal cell neurites.

## Discussion

Our results show that the complement protein C1q specifically regulates horizontal cell neurite confinement during development. Loss of C1q resulted in an increase in both the density and length of ectopic horizontal cell neurites. These defects were likely due to alterations in microglia-mediated neurite confinement. *C1q* expression was restricted to microglia during development, and *C1q^–/–^* mice showed reduced microglia activation. These results are the first to implicate the complement system generally and C1q specifically in outer retina development and suggest that C1q cannot only target particular synaptic lamina but can also achieve remarkable cellular specificity, removing neurites arising from one neuron type while sparing others present in the same synapse region.

### The Role of Complement in the Visual System

The complement system is well-known for its roles in disease-mediated immune activation both within and beyond the visual system where it can contribute to neuron loss. In the retina, C1q has been implicated in both age-related macular degeneration and glaucoma (Stasi et al., 2006; Anderson et al., 2010; Khandhadia et al., 2012; Ambati et al., 2013). In the brain C1q may participate in the progression of diseases ranging from epilepsy to Alzheimer’s disease (Eikelenboom and Stam, 1982; Ishii and Haga, 1984; Afagh et al., 1996; Pasinetti, 1996; Wyss-Coray et al., 2002; Fonseca et al., 2004; Aronica et al., 2007; Ma et al., 2013). Our results contribute to the body of evidence that suggests complement also regulates normal CNS development. In particular, complement has been shown to mediate removal of synapses and neurites at different relay points within the visual circuit. The first such discoveries were made using the lateral geniculate nucleus (LGN). In this system, deletion of C1q resulted in defects in eye specific segregation of retinogeniculate synapses in the dorsal lateral geniculate nucleus (dLGN) (Stevens et al., 2007). Loss of C1q resulted in increased overlap between retinogeniculate connections arising from the contralateral and ipsilateral eye, and a failure to refine these connections. However, both the refinement defects of the dLGN and the confinement defects of horizontal cells are not due to early development defects such as axon targeting. Further, both C1q dependent dLGN neurite remodeling and horizontal cell confinement seem to rely upon microglia to remove excess neural processes (Schafer et al., 2012). In addition, C1q appears to target a subset of RGCs for removal after they are born (Anderson et al., 2019). We now extend these findings to retinal neurite organization. To our knowledge, this study represents the first documented role for C1q in modulating neurite confinement in the retina.

### Precise Spatiotemporal Roles for Complement in the CNS

A key mystery in complement-mediated synapse refinement is whether this system is capable of not only regional activity but also cellular and perhaps subcellular specificity. Recent work suggests that C1q can indeed have regional roles in neurite refinement even within the visual system, as it is required in the LGN but appears dispensable in the primary visual cortex (Stevens et al., 2007; Welsh et al., 2020). The layered cellular and synapse organization in the retina enabled us to ask whether C1q specificity extended to individual cell types or to specific regions within a given neuron. This question is particularly tractable in the OPL because just four neuron types form synapses here in one ordered layer. We found that among these neuron types, only horizontal cells appear to require C1q for proper neurite organization. Further, only a subset of neurites arising from horizontal cells were targeted. Thus, even within a neuron type C1q dependent remodeling can achieve neurite-specific selectivity. Complement-mediated neurite targeting also displays unique temporal features. In both the outer retina and the dLGN, defects appear beginning in the second postnatal week. This time coincides with eye opening, in keeping with the idea that microglia participate in activity-dependent synapse elimination (Schafer et al., 2012). Taken together, these data suggest that complement mediated signaling may regulate subcellular confinement downstream of circuit activity.

### Production and Action of C1q in Neural Development

In this study, we asked two questions regarding C1q action: which cells are responsible for producing C1q, and how might C1q modulate horizontal cell organization? Our data point to a role for microglia. *C1q* levels were highest in the first two postnatal weeks when synapse refinement peaks and *C1q* mRNA was restricted to microglia during this time period. These results are consistent with other reports which found that complement proteins are locally synthesized by microglia and astrocytes in the CNS (Veerhuis et al., 1999; Veerhuis et al., 2003; Bialas and Stevens, 2013; Fonseca et al., 2017). Following its production, C1q can bind synapses and neurites in retina and in brain (Singhrao et al., 2000; Stevens et al., 2007). In turn, tagged synapses and neurites are thought to be removed by activated microglia (Schafer et al., 2012). Our data support this model. Microglia in C1q deficient retina showed decreased engulfment and activation, a finding that parallels that in C1q deficient dLGN (Schafer et al., 2012). However, it remains unclear which downstream signaling processes lead to C1q-dependent microglia-mediated engulfment in this and other systems. C1q can bind to a variety of receptors (Malhotra et al., 1990; Kishore et al., 1998; Kishore and Reid, 2000; Païdassi et al., 2008; Kouser et al., 2015). Once activated, C1q signaling can lead to the formation of the classical MAC via signaling through C3. However, in contrast to complement-mediated damage in neural disease (Lian et al., 2016; Czir et al., 2017; Litvinchuk et al., 2018), we find that horizontal cell neurite confinement is independent of both C3aR and CR3. C1q can also induce several MAC-independent pathways, including canonical WNT signaling (Korb and Ahearn, 1997; Ogden et al., 2001; Païdassi et al., 2008; Hong et al., 2009; Naito et al., 2012; Bossi et al., 2014). Additionally, complement receptors (*cr2, gc1qr*, *cc1qr*) and regulators (*vtn*, *clu*, *crry*) have been found to be expressed in horizontal cells (Pauly et al., 2019). In future studies, it will be interesting to determine whether complement-dependent neural remodeling functions through these distinct mechanisms in development.

Together our data demonstrate a new role for complement-dependent neurite remodeling in the developing outer retina. Of note is the finding that this system can function with both neuron-type dependent specificity and subcellular specificity. This suggests additional levels of regulation that could be distinct in different neuron types or compartments. Such precise neurite remodeling may also reflect potential roles for distinct sub-populations of microglia (Hammond et al., 2019; Li Q. et al., 2019). The retina will continue to be an important tool for resolving these and related mysteries.

## Data Availability Statement

The raw data supporting the conclusions of this article will be made available upon reasonable request.

## Ethics Statement

The animal study was reviewed and approved by Baylor College of Medicine Institutional Animal Care and Use Committee.

## Author Contributions

CB, DJ, and MS designed the experiments and wrote the manuscript. FL conducted the primary analysis of the mutant mice. CB, DJ, and FL acquired the images. DJ performed IMARIS reconstructions and RNAscope and qRT-PCR. CB and DJ conducted the histological data analyses and generated the figures. CB performed ERG and viral injections. All authors contributed to the article and approved the submitted version.

## Conflict of Interest

The authors declare that the research was conducted in the absence of any commercial or financial relationships that could be construed as a potential conflict of interest.

## Supplementary Material

Supplementary Figure 1 Loss of C1q leads to areduction in retinal function. **(A)** Representative traces of scotopic recordings from wild type control and *C1q^–/–^* mice. The scotopic a-wave **(B)** and b-wave **(C)** are significantly reduced in *C1q^–/–^* mice. *N* = 4 wild type control and 4 *C1q^–/–^* mice. The implicit time for scotopic a-wave **(D)** and scotopic b-wave **(E)** is not significantly different. *N* = 4 wild type control and 4 *C1q^–/–^* mice. The photopic a-wave **(F)** is lower but not significantly reduced in *C1q^–/–^* mice while the photopic b-wave **(G)** is significantly decreased. *N* =4 wild type control and 3 *C1q^–/–^* mice. Data are represented as the mean ± SEM. ^∗∗∗^*p* < 0.001, ^∗∗^*p* < 0.01, ^∗^*p* < 0.05, unpaired two-tailed Student’s *t-*test.

Supplementary Figure 2 Loss of *C1q* mRNA in C1q knockout mice. **(A)** Representative fluorescent *in situ* hybridization image of *C1q* (green) in *C1q^–/–^* mice at P13. **(B)** qRT-PCR for *C1q* mRNA in *C1q^–/–^* and control mice at P13. Values represent the fold mRNA expression level relative to the levels detected in control animals following normalization to *GAPDH*. There is a significant decrease in the levels of *C1q* mRNA in *C1q^–/–^* mice compared to wild type controls. *N* = 3 wild type control and 3 *C1q^–/–^* mice. Scale bars = 25 μm. Data are represented as the mean ± SEM. ^∗∗∗^*p* < 0.001, unpaired two-tailed Student’s *t-*test.

Supplementary Figure 3 Synapse layer emergence appears normal in *C1q^–/–^* animals. **(A)** Representative images of the OPL (arrows) whose upper boundary is defined by cells in the ONL (nuclei, DAPI) and whose lower boundary is defined by cells in the INL (nuclei, DAPI; horizontal cells bodies, stars) at P5 and P8. The OPL emerges normally in both wild type control and *C1q^–/–^* mice. **(B)** Representative images of horizontal cells (calbindin) at P5. Refinement of horizontal cell neurites in both wild type control and *C1q^–/–^* mice appears normal. **(C)** Representative images of cones (OPN1SW) at P5. Axon extension in both wild type control and *C1q^–/–^* mice appears normal. Scale bars = 25 μm.

Supplementary Figure 4 Loss of C1q does not alter horizontal cell mosaic spacing. **(A)** Representative flat mount images of horizontal cells (calbindin, cyan) in control and *C1q^–/–^* mice at P10. Quantifications of horizontal cell density **(B)**, Voronoi domain regularity index **(C)**, and density recovery profiles of control and *C1q^–/–^* horizontal cells **(D)**. There is no significant difference in horizontal cell density or spacing. *N* = 3 wild type control and 3 *C1q^–/–^* mice. Scale bars = 25 μm. Data are represented as the mean ± SEM.

Supplementary Figure 5 Loss of C1q does not alter the morphological features of other outer retina neuron types. **(A)** Representative images of cones (Arrestin, red; P17), cone bipolar cells (secretagogin, magenta; P13), and rod bipolar cells (PKCα, green; P13) in wild type control and *C1q^–/–^* mice. No apparent changes in cellular organization or neurite morphology are observed in these cells. **(B)** Quantifications of retinal layer thickness in wild type control and *C1q^–/–^* mice across development. No significant difference was observed in layer thickness at P13, P17, or P23. *N* ≥ 3 mice for each time point. Scale bars = 25 μm. Data are represented as the mean ± SEM.

Supplementary Figure 6 Organization and levels of presynaptic proteins in the outer retina are not altered with the loss of C1q. **(A)** Representative images of synapse-associated proteins PSD95 (yellow), RIBEYE (magenta), and VGLUT1 (green) in wild type control and *C1q^–/–^* mice at P13. No changes in organization of these synapse-associated proteins are observed. **(B)** Representative images of synapse-associated proteins PSD95 (yellow), RIBEYE (magenta), and VGLUT1 (green) in wild type control and *C1q^–/–^* mice at P17. No changes in organization of these synapse-associated proteins are observed. **(C)** qRT-PCR for synaptic proteins *PSD95*, *vesicular glutamate transporter 1* (*VGlut1*), *Synaptotagmin2* (*Syt2*), and *Synaptophysin* (*SYP*) at P13 in wild type control and *C1q^–/–^* mice. Values represent the fold mRNA expression level relative to the levels detected in control animals following normalization to *GAPDH*. There is no significant difference in levels of mRNA between wild type control and *C1q^–/–^* mice. Scale bars = 25 μm. Data are represented as the mean ± SEM.

Supplementary Figure 7 Microglia morphology is not significantly altered in the outer retina in the absence of C1q. **(A)** Representative images of microglia (Iba1, magenta) localization in distinct retina layers as visualized by co-staining for nuclei (DAPI, gray) at P10. No apparent differences were observed in microglia localization in *C1q^–/–^* animals relative to wild type controls. **(B)** Representative flat mount images of microglia (Iba1, magenta) in the OPL in wild type control and *C1q^–/–^* mice at P10. Quantifications of the number of microglia process endpoints per cell **(C)** and the summed branch length per microglia **(D)** in wild type control and *C1q^–/–^* mice at P10. No significant differences were observed in either parameter. *N* = 3 control and *N* = 3 *C1q^–/–^* mice. Scale bars = 25 μm. Data are represented as the mean ± SEM.

Supplementary Video 1 Dendrites of a wild type control horizontal cell.

Supplementary Video 2 Axon and axon terminal of a wild type control horizontal cell.

Supplementary Video 3 Dendrites of C1q knockout horizontal cell 1.

Supplementary Video 4 Axon and axon terminal of C1q knockout horizontal cell 1.

Supplementary Video 5 Dendrites of C1q knockout horizontal cell 2.

Supplementary Video 6 Axon and axon terminal of C1q knockout horizontal cell 2.
