# Supplementary figures and images for: C1q Regulates Horizontal Cell Neurite Confinement in the Outer Retina
Source: Front Neural Circuits. 2020 Oct 16;14:583391. doi: 10.3389/fncir.2020.583391 (PMC7596380; doi:10.3389/fncir.2020.583391)

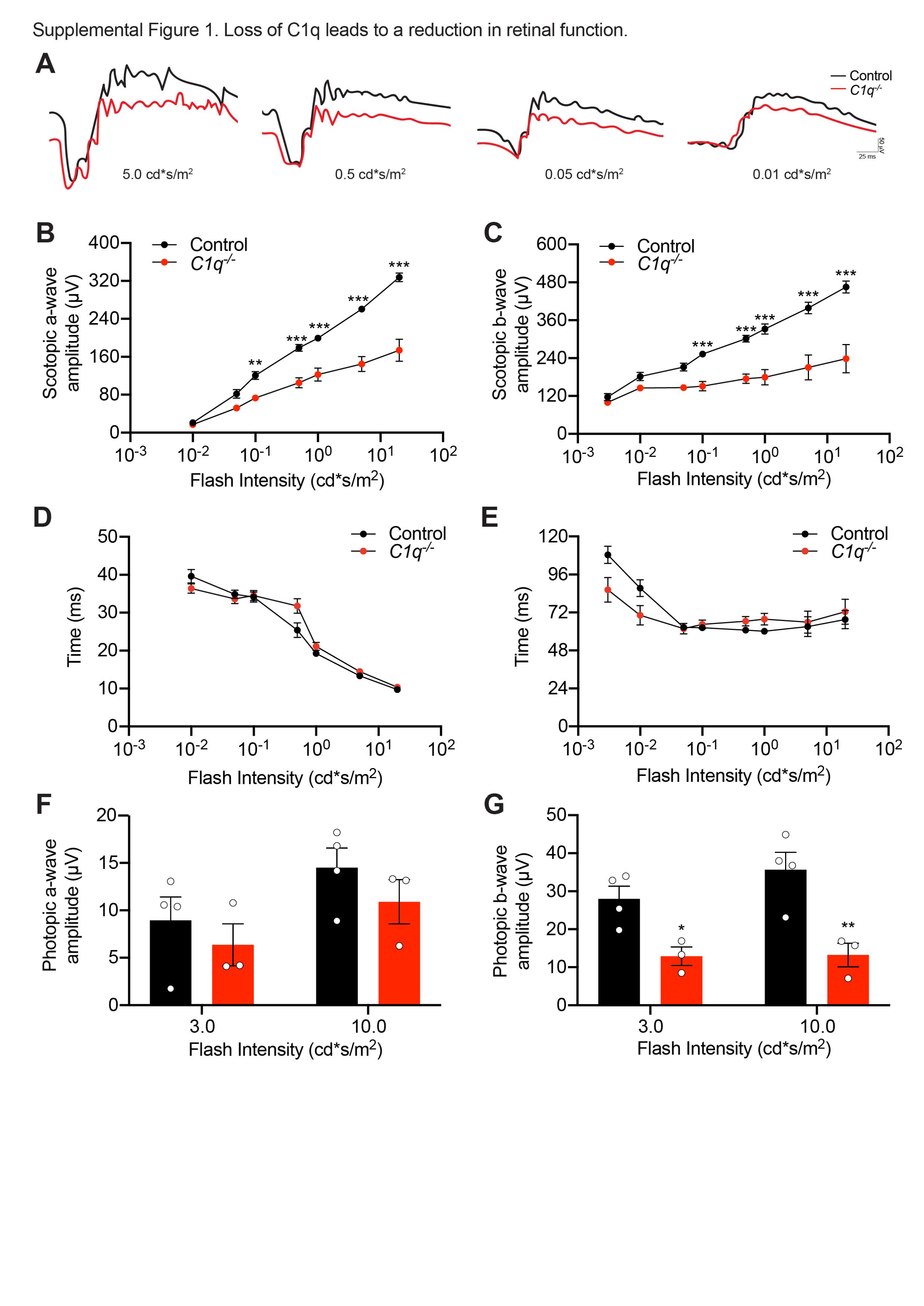

Supplement: Supplementary Figure 1 — Loss of C1q leads to areduction in retinal function. (A) Representative traces of scotopic recordings from wild type control and C1q–/– mice. The scotopic a-wave (B) and b-wave (C) are significantly reduced in C1q–/– mice. N = 4 wild type control and 4 C1q–/– mice. The implicit time for scotopic a-wave (D) and scotopic b-wave (E) is not significantly different. N = 4 wild type control and 4 C1q–/– mice. The photopic a-wave (F) is lower but not significantly reduced in C1q–/– mice while the photopic b-wave (G) is significantly decreased. N =4 wild type control and 3 C1q–/– mice. Data are represented as the mean ± SEM. ∗∗∗p < 0.001, ∗∗p < 0.01, ∗p < 0.05, unpaired two-tailed Student’s t-test. [file Image_1.jpg]

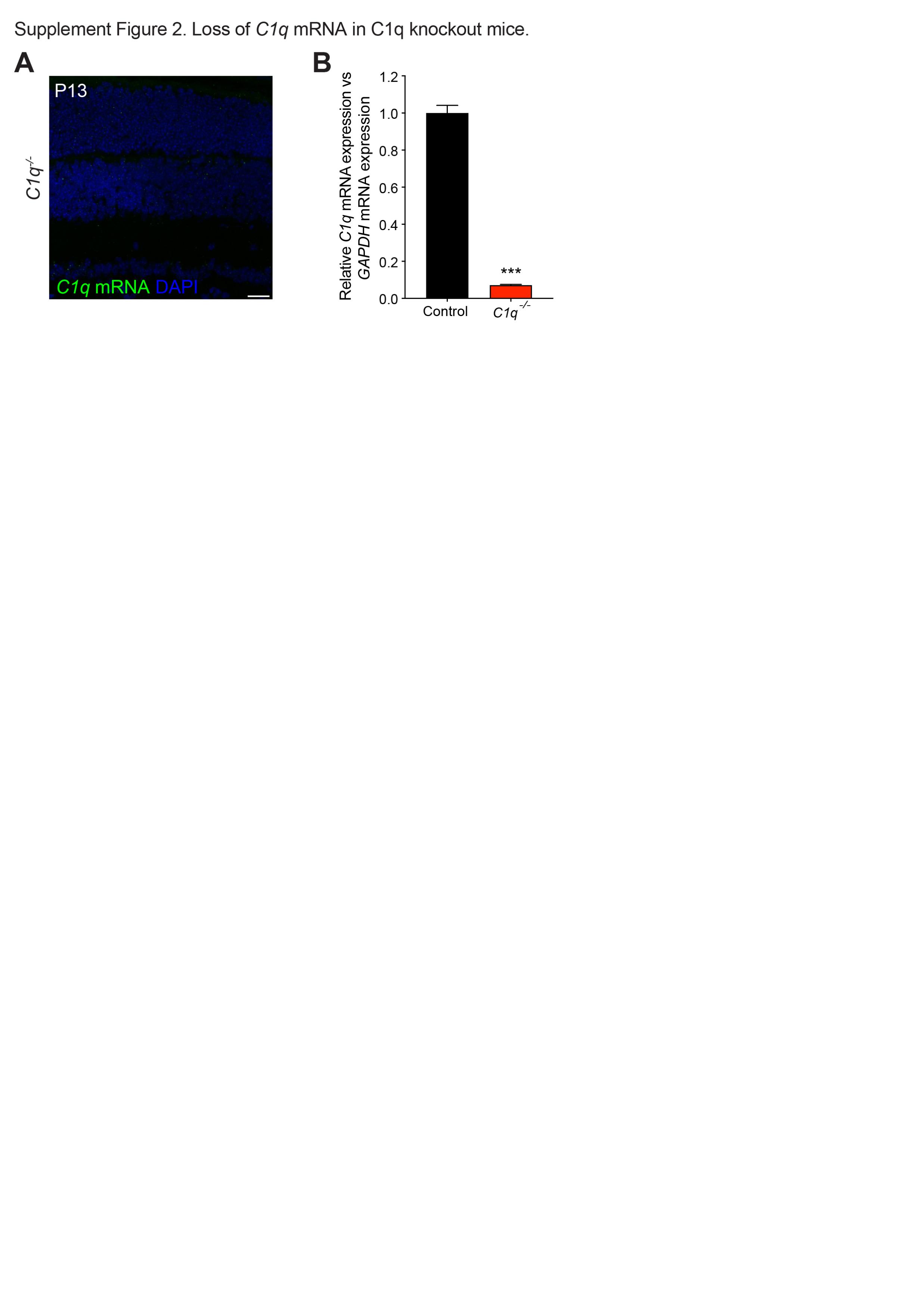

Supplement: Supplementary Figure 2 — Loss of C1q mRNA in C1q knockout mice. (A) Representative fluorescent in situ hybridization image of C1q (green) in C1q–/– mice at P13. (B) qRT-PCR for C1q mRNA in C1q–/– and control mice at P13. Values represent the fold mRNA expression level relative to the levels detected in control animals following normalization to GAPDH. There is a significant decrease in the levels of C1q mRNA in C1q–/– mice compared to wild type controls. N = 3 wild type control and 3 C1q–/– mice. Scale bars = 25 μm. Data are represented as the mean ± SEM. ∗∗∗p < 0.001, unpaired two-tailed Student’s t-test. [file Image_2.jpg]

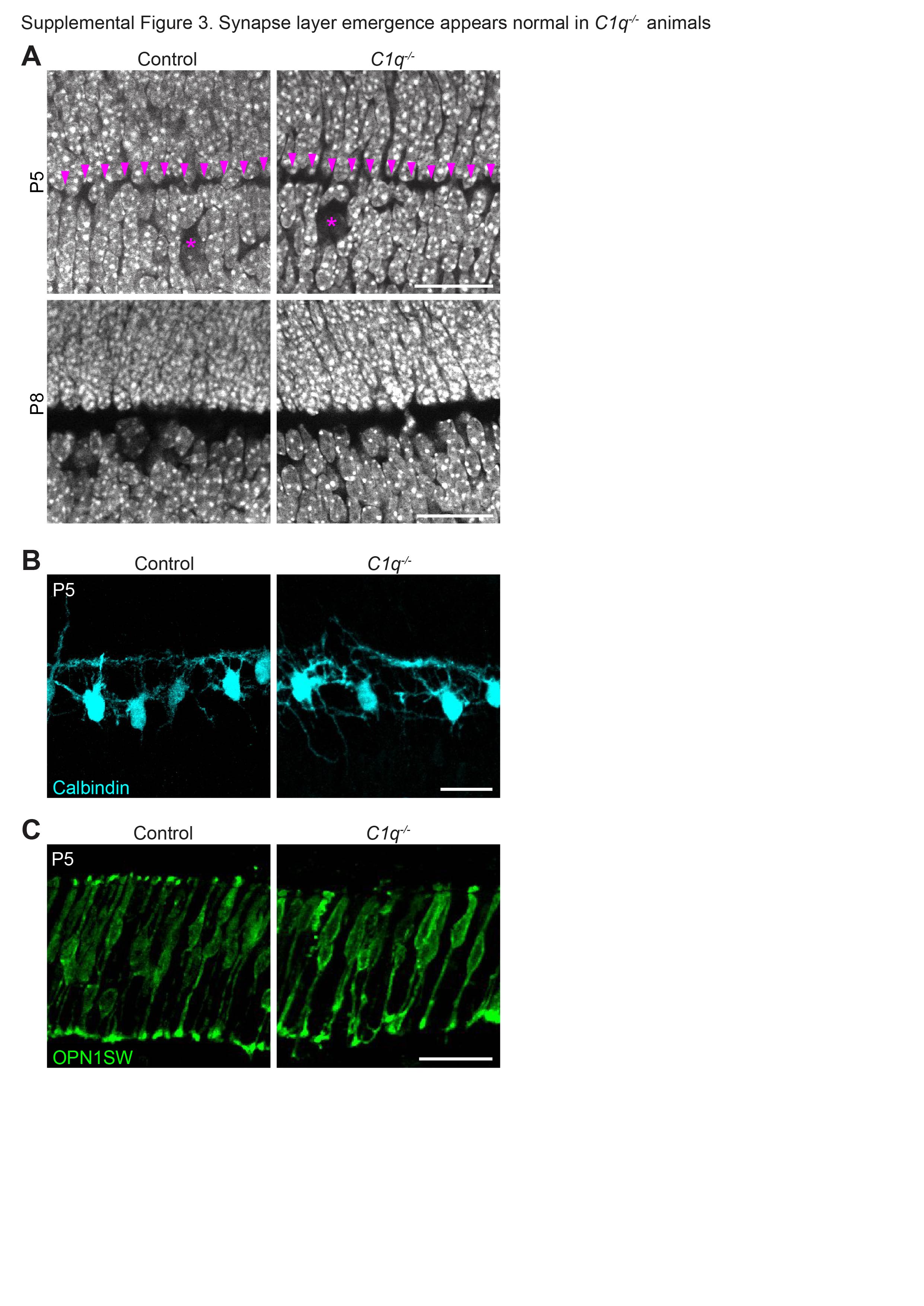

Supplement: Supplementary Figure 3 — Synapse layer emergence appears normal in C1q–/– animals. (A) Representative images of the OPL (arrows) whose upper boundary is defined by cells in the ONL (nuclei, DAPI) and whose lower boundary is defined by cells in the INL (nuclei, DAPI; horizontal cells bodies, stars) at P5 and P8. The OPL emerges normally in both wild type control and C1q–/– mice. (B) Representative images of horizontal cells (calbindin) at P5. Refinement of horizontal cell neurites in both wild type control and C1q–/– mice appears normal. (C) Representative images of cones (OPN1SW) at P5. Axon extension in both wild type control and C1q–/– mice appears normal. Scale bars = 25 μm. [file Image_3.jpg]

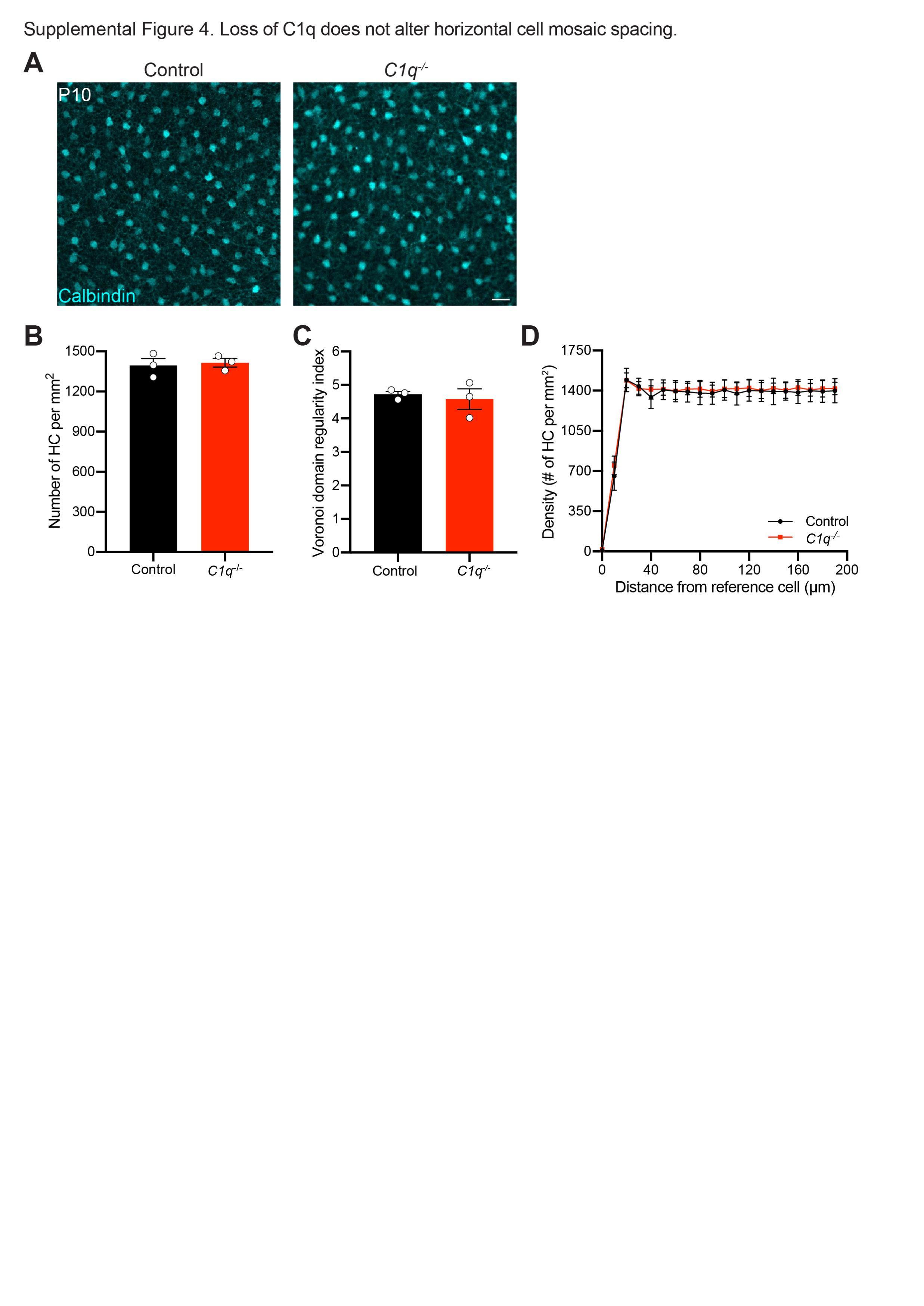

Supplement: Supplementary Figure 4 — Loss of C1q does not alter horizontal cell mosaic spacing. (A) Representative flat mount images of horizontal cells (calbindin, cyan) in control and C1q–/– mice at P10. Quantifications of horizontal cell density (B), Voronoi domain regularity index (C), and density recovery profiles of control and C1q–/– horizontal cells (D). There is no significant difference in horizontal cell density or spacing. N = 3 wild type control and 3 C1q–/– mice. Scale bars = 25 μm. Data are represented as the mean ± SEM. [file Image_4.jpg]

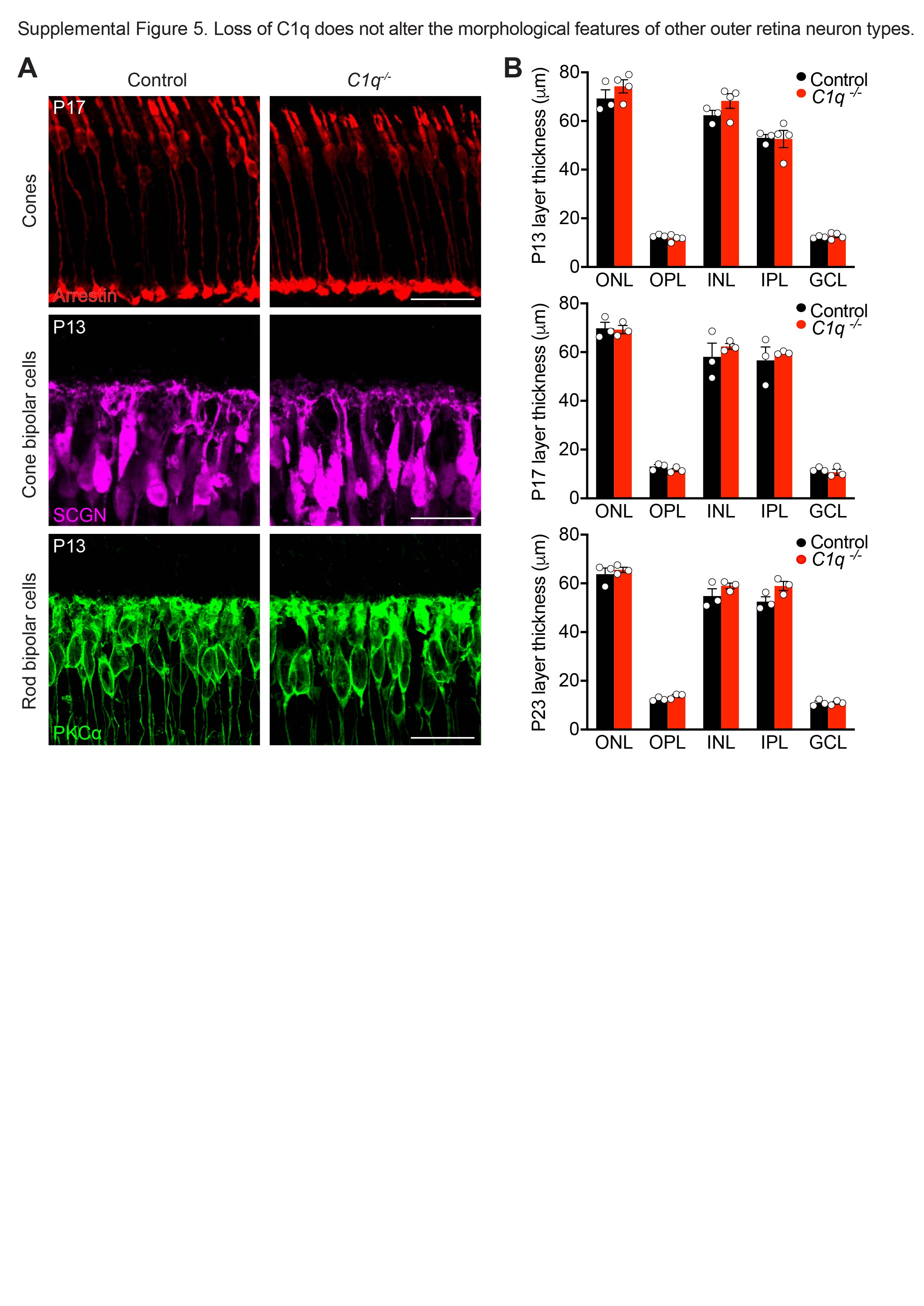

Supplement: Supplementary Figure 5 — Loss of C1q does not alter the morphological features of other outer retina neuron types. (A) Representative images of cones (Arrestin, red; P17), cone bipolar cells (secretagogin, magenta; P13), and rod bipolar cells (PKCα, green; P13) in wild type control and C1q–/– mice. No apparent changes in cellular organization or neurite morphology are observed in these cells. (B) Quantifications of retinal layer thickness in wild type control and C1q–/– mice across development. No significant difference was observed in layer thickness at P13, P17, or P23. N ≥ 3 mice for each time point. Scale bars = 25 μm. Data are represented as the mean ± SEM. [file Image_5.jpg]

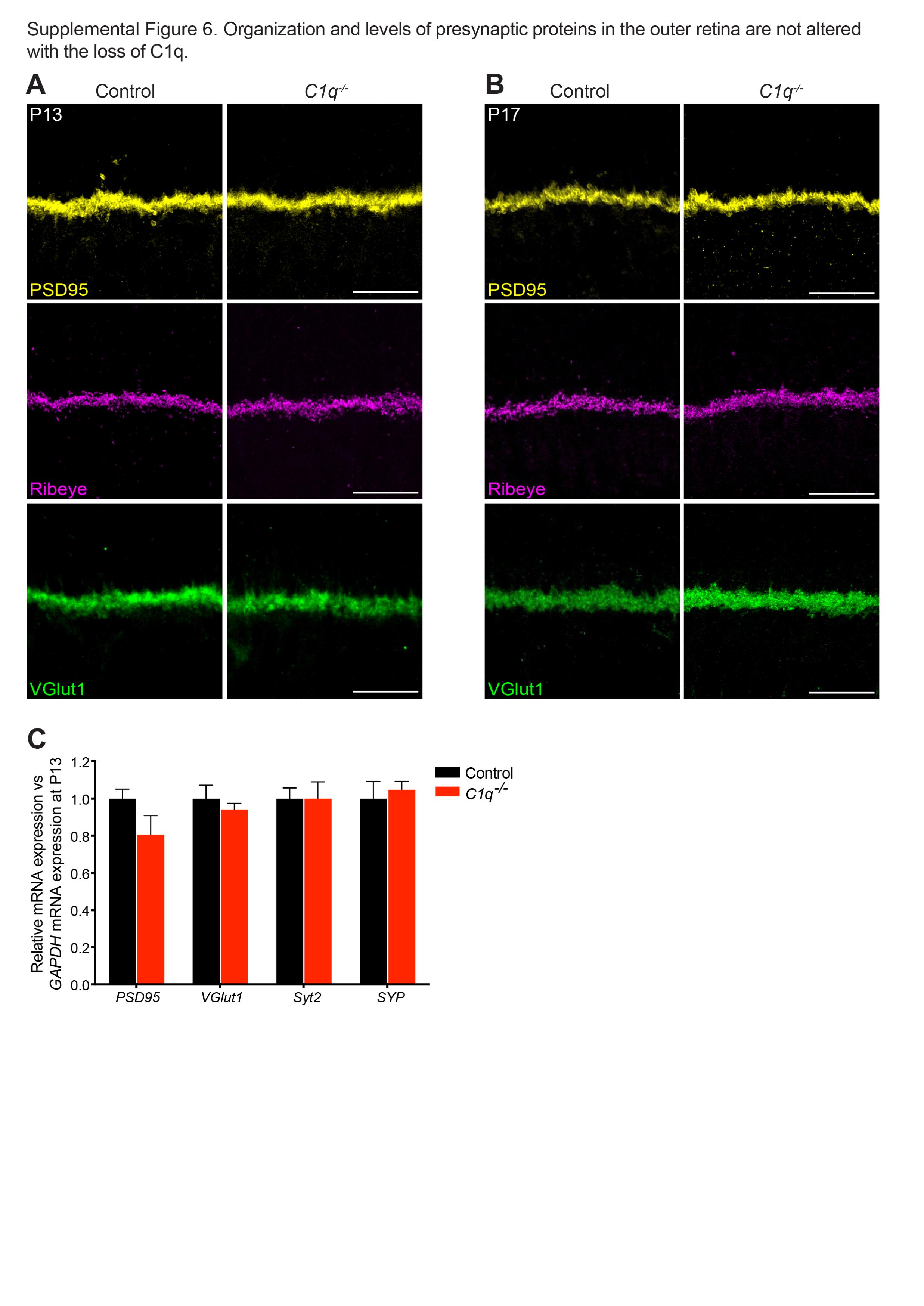

Supplement: Supplementary Figure 6 — Organization and levels of presynaptic proteins in the outer retina are not altered with the loss of C1q. (A) Representative images of synapse-associated proteins PSD95 (yellow), RIBEYE (magenta), and VGLUT1 (green) in wild type control and C1q–/– mice at P13. No changes in organization of these synapse-associated proteins are observed. (B) Representative images of synapse-associated proteins PSD95 (yellow), RIBEYE (magenta), and VGLUT1 (green) in wild type control and C1q–/– mice at P17. No changes in organization of these synapse-associated proteins are observed. (C) qRT-PCR for synaptic proteins PSD95, vesicular glutamate transporter 1 (VGlut1), Synaptotagmin2 (Syt2), and Synaptophysin (SYP) at P13 in wild type control and C1q–/– mice. Values represent the fold mRNA expression level relative to the levels detected in control animals following normalization to GAPDH. There is no significant difference in levels of mRNA between wild type control and C1q–/– mice. Scale bars = 25 μm. Data are represented as the mean ± SEM. [file Image_6.jpg]

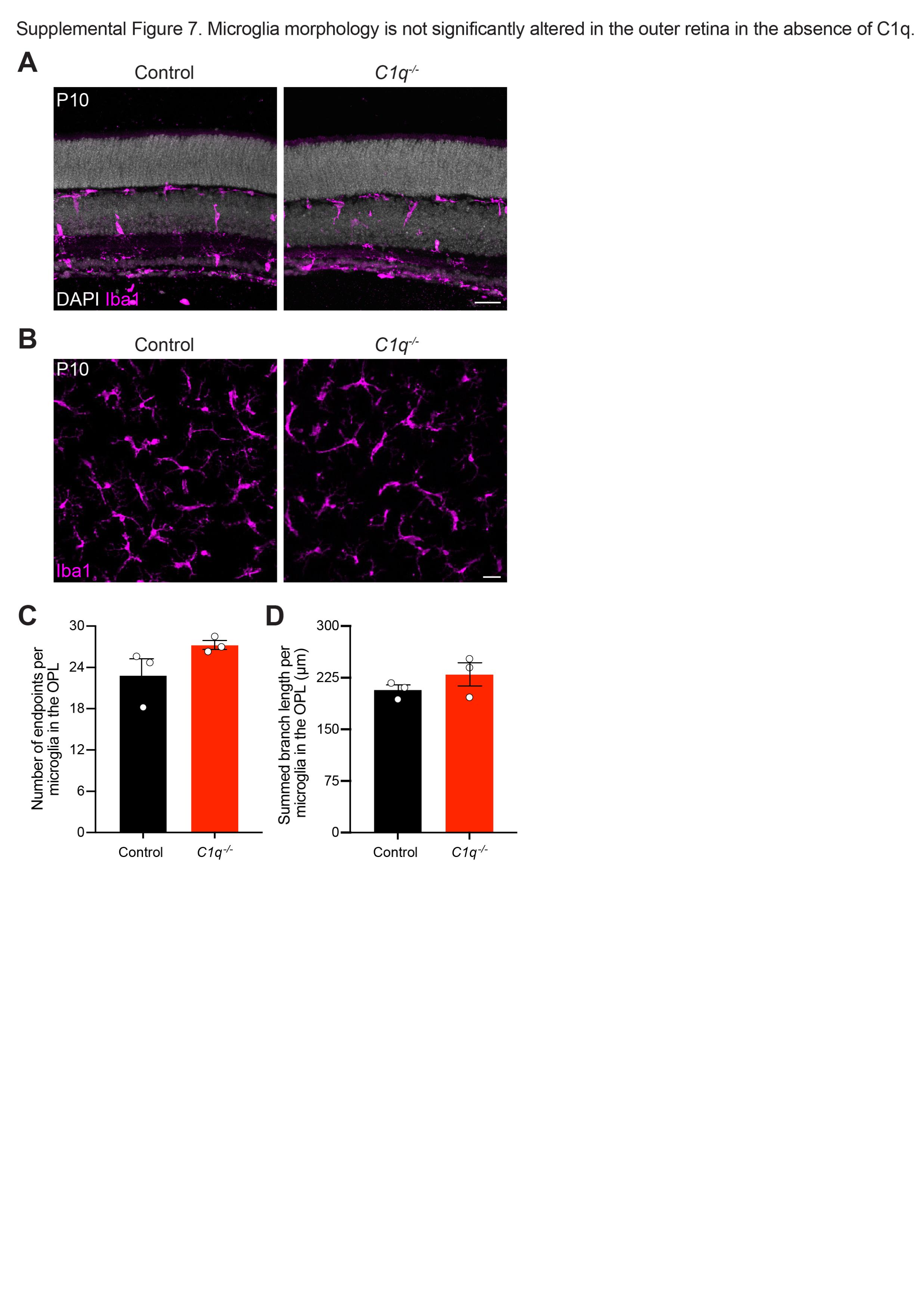

Supplement: Supplementary Figure 7 — Microglia morphology is not significantly altered in the outer retina in the absence of C1q. (A) Representative images of microglia (Iba1, magenta) localization in distinct retina layers as visualized by co-staining for nuclei (DAPI, gray) at P10. No apparent differences were observed in microglia localization in C1q–/– animals relative to wild type controls. (B) Representative flat mount images of microglia (Iba1, magenta) in the OPL in wild type control and C1q–/– mice at P10. Quantifications of the number of microglia process endpoints per cell (C) and the summed branch length per microglia (D) in wild type control and C1q–/– mice at P10. No significant differences were observed in either parameter. N = 3 control and N = 3 C1q–/– mice. Scale bars = 25 μm. Data are represented as the mean ± SEM. [file Image_7.jpg]
